# People With Non‐Communicable Diseases Using Ayurveda: A UK‐Based Qualitative Study

**DOI:** 10.1111/hex.70494

**Published:** 2025-11-18

**Authors:** Patricia Egwumba, Kaushik Chattopadhyay, Laura Nellums, Manpreet Bains

**Affiliations:** ^1^ Lifespan and Population Health, School of Medicine University of Nottingham UK; ^2^ The Nottingham Centre for Evidence‐Based Healthcare: a JBI Centre of Excellence Nottingham UK; ^3^ College of Population Health, Health Sciences Centre University of New Mexico Albuquerque New Mexico USA

**Keywords:** Ayurveda, disease management, non‐communicable diseases, qualitative research, United Kingdom

## Abstract

**Introduction:**

Non‐communicable diseases (NCDs) are a leading cause of morbidity and mortality in the United Kingdom, placing significant pressure on the National Health Service (NHS). Despite the growing popularity of Ayurveda for managing NCDs, little is known about its use among people with these conditions in the United Kingdom. This study explored the experiences and perspectives of people with NCDs who use Ayurveda to manage their conditions in the United Kingdom.

**Methods:**

Twenty qualitative semi‐structured interviews were conducted with UK‐based adults with NCDs. Interviews were audio‐recorded, transcribed verbatim and analysed using thematic analysis.

**Findings:**

Three key themes were identified. First, participants chose Ayurveda due to its alignment with personal values like natural, holistic healing and dissatisfaction with Western medicine, particularly side effects and impersonal care. Second, they reported positive experiences with Ayurvedic treatment, including personalised consultations, diverse treatment options and improved health outcomes. Third, participants highlighted challenges in sustaining Ayurvedic care, such as concerns over product safety, difficulty following complex regimens, limited medicine availability and financial barriers—especially since treatments are not covered by the NHS.

**Conclusion:**

People living with NCDs described Ayurveda as a more natural and philosophically congruent healing system, reflecting their cultural and personal perspectives. Despite structural and financial challenges, they considered it a relevant option for managing their conditions. These findings suggest that Ayurveda continues to hold significance as a complementary approach to NCD management in the United Kingdom.

## 1 Introduction

Non‐communicable diseases (NCDs) are conditions that are not transmitted from person to person; however, they typically have a long duration and slow progression [1, 2]. In the United Kingdom, NCDs are one of the largest contributors to the disease burden in the National Health Service (NHS) [3, 4, 5], with NCDs responsible for an estimated 89% of all years lived with disability (YLDs), 81% disability‐adjusted life years (DALYs) [6]. Similarly, NCDs are the leading cause of death in the United Kingdom. In 2019, 89% of total deaths were attributed to NCDs, which is higher than the global average of 74% [6]. NCDs remain a central challenge for patients and the healthcare system and are therefore a major concern for the NHS [7]. As the prevalence of NCDs continues to rise, there is growing interest in more holistic and individualised approaches to NCD treatment, such as complementary and alternative medicine (CAM) [8, 9, 10, 11, 12, 13]. Approximately 44% of individuals in the United Kingdom have used CAM in their lifetime [14], with most accessing via the private sector [8]. Socio‐demographic patterns also show that CAM use in the United Kingdom is positively associated with higher income levels, white‐collar occupations and attainment of higher education qualifications [8].

Motivations for using CAM are varied and include dissatisfaction with conventional care, a desire to avoid pharmaceutical side effects, and a preference for natural or traditional healing methods [8, 9, 10, 12]. Trends in access to complementary medicines show that CAM has been accessed by a wide range of NHS priority groups, including individuals with cancer (11%), elderly patients with various types of musculoskeletal conditions (10%), those with mental health concerns (9%), and those managing diabetes or coronary heart disease (5%) [9]. This pattern reflects a growing role for CAM in the management of NCDs, particularly where conventional care may not fully meet patients' needs. CAM is often perceived as aligning with individuals' broader values, beliefs and preferences around health and well‐being [8, 9, 10, 11, 12].

Among the various CAM approaches available, Ayurveda, a traditional system of medicine with roots in the Indian subcontinent, offers a comprehensive approach that uses Ayurvedic detoxifying and purifying therapies (e.g., Panchakarma) and Ayurvedic medicines (containing plant‐, animal‐ or mineral‐origin ingredients—single or in combination) [15, 16]. While Ayurveda is globally recognised and utilised, very few empirical studies have explored how and why individuals with NCDs in the United Kingdom turn to Ayurveda for managing their conditions [10]. While prior UK‐based studies have explored the perspectives of Ayurvedic practitioners [17, 18], there remains a notable absence of qualitative research focusing specifically on patients' experiences and perspectives on using Ayurveda to manage their conditions. This study explored the experiences and perspectives of people with NCDs regarding the use of Ayurveda for managing their conditions.

## 2 Methods

### 2.1 Study Design, Ethics and Reporting

This qualitative research was guided by a constructivist philosophical paradigm and was conducted in the United Kingdom in 2023, employing a descriptive exploratory design [19, 20]. The study was approved by the University of Nottingham's Faculty of Medicine and Health Sciences Research Ethics Committee (FMHS 298‐0523). Reporting followed the Consolidated Criteria for Reporting Qualitative Research (COREQ‐32) guidelines [21].

### 2.2 Recruitment and Sampling

Adults were eligible to participate if they lived in the United Kingdom, had a diagnosed NCD, and had been using Ayurvedic treatment for a minimum of 6 months. Purposive sampling was used to identify the participants. UK‐based Ayurvedic practitioners were asked to assist with recruitment by circulating a study poster containing the contact details of the lead researcher (P.E.) among their patients. Eligible participants were encouraged to refer others who met the inclusion criteria. A total of 23 individuals expressed interest in participating in the study and were given an information sheet explaining the purpose of the study, confidentiality and the process of obtaining informed consent. All participants provided written informed consent before participating in the interviews. Guidance for qualitative studies employing semi‐structured interviews indicates that approximately 20 interviews are typically sufficient to achieve thematic saturation [19, 20]. In this study, saturation was reached by the 16th interview; however, data collection was continued until the 20th interview to ensure that no relevant insights were missed.

### 2.3 Interview Guide and Data Collection

The research team developed an interview guide using the study's objective and relevant literature. Probing and open‐ended questions were incorporated into the guide to encourage participants to openly express their opinions [19, 20]. The guide was piloted with two participants, and their data were included in the final analysis, as no revisions were necessary. All interviews were conducted in English by P.E. using the guide and Microsoft Teams. Interviews were audio‐recorded with consent. The process and purpose of the study were reiterated to participants before each interview.

### 2.4 Data Analysis and Reflexivity

The lead researcher transcribed two interview recordings verbatim, while the remaining recordings were transcribed by an external transcription company under a non‐disclosure agreement. To ensure accuracy, the lead researcher cross‐checked the transcripts against the audio files and anonymised any identifiable information. Transcripts were imported into NVivo 14.0 software to support data organisation and analysis [22].

Data were analysed following Braun and Clarke's thematic analysis approach [23]. P.E. read through the transcripts multiple times for familiarisation, followed by inductive, line‐by‐line coding. To enhance credibility and dependability, we employed investigator triangulation: multiple team members were involved in coding, reviewing and refining themes, and any differences in interpretation were discussed until consensus was reached [19, 24]. The lead researcher subsequently organised sub‐themes into broader themes, which were checked collaboratively to ensure they corresponded with the coded extracts. Participant quotations were anonymised and labelled as ‘P’ (participant), with verbatim quotes used except where ellipses (…) indicated minor omissions for clarity and readability.

The analysis was conducted using a fundamentally reflexive approach. As a trained Western medicine practitioner, P.E. recognised that she had no direct experience with people who use Ayurveda to manage NCDs and was aware of the influence of positionality on interpretation. To address this, the lead researcher analysed data inductively, focusing on participants' own language and meanings in coding and cross‐checked interpretations against the cultural contexts raised in the interviews. Reflexive journaling and collaborative reflexivity (among the team) throughout the study process and repeated review of transcripts helped minimise bias and ensure accurate representation of participant perspectives.

The research team consisted of academics from the University of Nottingham with complementary expertise relevant to the study. The lead researcher, P.E., is a PhD student who conducted interviews, led data collection and coding, and drafted the manuscript. K.C., Associate Professor and a registered medical doctor trained in Ayurveda, supervised the project, contributed to the study design, validated the analysis and reviewed the manuscript. M.B., Associate Professor and experienced qualitative researcher, provided guidance on coding and theme development and contributed to manuscript review.

## 3 Results

### 3.1 Participant Characteristics

We interviewed 20 participants; the average duration of the interviews was 44 min (range: 20–68 min). The characteristics of the participants included in this study are presented in Table 1. The average age of the participants was 59 years, and most had been using Ayurveda for over 6 years. The sample was predominantly female and British Asian. Most participants were employed and held a first (bachelor's) degree, with some having additional qualifications such as a master's or PhD. The group was religiously heterogeneous, with most participants identifying as atheists and Hindus. Most participants reported using Ayurveda for the treatment of several NCDs, including musculoskeletal, gastrointestinal, cardiovascular and autoimmune conditions.

**Table 1:** Characteristics of participants.

Characteristic	*N* (%)
Age (years), Mean ± SD	59 ± 12
Sex	
Female	12 (60%)
Male	8 (40%)
Ethnicity	
White British	7 (35%)
British Asian	13 (65%)
Highest education	
High school	1 (5%)
Bachelor's degree	11 (55%)
Master's degree	5 (25%)
PhD	3 (15%)
Employment status	
Retired	5 (25%)
Employed	15 (75%)
Religion	
Hindu	7 (35%)
Atheist	9 (45%)
Muslim	3 (15%)
Christian	1 (5%)
Underlying disease condition	
Autoimmune	2 (10%)
Musculoskeletal	4 (20%)
Gastrointestinal	3 (15%)
Cardiovascular	5 (25%)
Other	6 (30%)
Duration of Ayurveda use	
0–5 years	4 (20%)
6–20 years	10 (50%)
> 20 years	6 (30%)

### 3.2 Qualitative Themes

Three key themes with corresponding sub‐themes were identified through thematic analysis of the qualitative data, as presented in Table 2.

**Table 2:** Themes and sub‐themes identified.

Themes	Sub‐themes
1. Motivations for choosing Ayurveda for NCD management	1.1 Personal, cultural and philosophical influences
	1.2 Dissatisfaction with conventional Western medicine
	1.3 Trust and practitioner selection
4. Experiences and perceived benefits of Ayurvedic care	2.1 Holistic and individualised consultations
	2.2 Integration of Western diagnostics
	2.3 Treatment practices and preferences
	2.4 Perceived health improvements
8. Challenges and concerns in accessing and sustaining Ayurvedic care	3.1 Safety concerns and product quality
	3.2 Adherence to Ayurvedic medicines
	3.3 Difficulty in sourcing Ayurvedic medicines for NCD management
	3.4 Financial challenges in accessing Ayurvedic care for NCDs

#### 3.2.1 Motivations for Choosing Ayurveda for NCD Management

This theme explored the underlying reasons why people with NCDs choose Ayurveda as a primary or secondary choice of care. These motives were based on personal values, dissatisfaction with Western medical care, and cultural, familial or social experience with Ayurvedic practice.

##### 3.2.1.1 Personal, Cultural and Philosophical Influences

Participants revealed that they were often inspired by the positive experience of family members who had successfully used Ayurveda to manage NCDs. Several participants explained that such success stories reassured and motivated them to explore Ayurvedic care themselves. Alongside familial influence was their exposure to yoga, meditation and other holistic methods; these also fostered their interest in Ayurveda. For many participants, Ayurveda's connection with the body's own healing mechanism and its philosophy of self‐repair made it more appealing. Cultural congruence also largely contributed, as participants perceived Ayurveda as more in line with their personal values, cultural background, philosophical worldviews and beliefs about health and healing.

‘My dad's heritage is Indian; I have a connection to India, I know Ayurveda works, and I believe in its healing benefits, especially for NCDs … we thought it would be interesting, so I went to India, on a visit, and we went to an Ayurvedic practitioner over there, and then a lot of stuff that they said made sense … I just came back to the UK, and have always had an Ayurvedic doctor in the UK’.

P9

##### 3.2.1.2 Dissatisfaction With Conventional Western Medicine

There was disillusionment with the Western medical treatment approach for managing NCDs. Some participants described years of escalating visits to Western‐trained specialists and long‐term use of prescription medications that failed to alleviate their symptoms. Many also expressed feeling unheard and unsupported in their care. Some participants explicitly mentioned suffering from the side effects of conventional Western medications while treating various NCDs. One participant who suffered severe reactions to thyroxine mentioned experiencing relief through Ayurveda's gentler healing approach.

‘About 3 years ago, I started on thyroxine … the side effects were horrible—swollen ankles, coughing, and thumping headaches—even on a very low dose…. In contrast, Ayurvedic medicine is different; I trust herbal remedies because I did not have side effects’.

P2

Some participants mentioned that after being informed by a Western medical practitioner that their conditions were incurable, they turned to Ayurveda as a last resort. For most participants, Ayurveda presented itself not simply as an alternative for managing NCDs but as a system that promises the hope of healing.

‘I was ill for quite a long time with Meniere's disease … there's no cure, and there's no tablets or anything … so when my friend told me about the treatment that she got from Dr A, that's when I decided I thought I would try it’.

P4

##### 3.2.1.3 Trust and Practitioner Selection

Participants mentioned that Ayurvedic practitioners' credibility played a key role in their decision to enrol in an Ayurvedic treatment plan. Participants selected Ayurvedic practitioners mainly based on their competence in managing NCDs. The number of NCD cases successfully handled by an Ayurvedic practitioner was, for many, a key metric of competence. Participants were also influenced by online content; for instance, some participants mentioned visiting online sites, such as Ayurvedic practitioners' websites and social media sites, to locate practitioners who could treat their condition. Other participants monitored patient reviews; these participants explained that patient testimonials and case studies mostly contributed to their decision‐making and instilled a feeling of trust and confidence in the practitioner's abilities.

‘One of them I came across had a very good Instagram account with a lot of in‐depth free content, and so I thought they would be very thorough, high‐quality, kind of very in‐depth, and experienced in managing NCDs’.

P1

#### 3.2.2 Experiences and Perceived Benefits of Ayurvedic Care

This theme highlighted participants' experiences with Ayurvedic care, focusing on consultation quality, treatment approaches, integration of Western diagnostics, and perceived benefits of Ayurvedic care.

##### 3.2.2.1 Holistic and Individualised Consultations

Participants emphasised that Ayurvedic consultations for NCDs were personalised, in‐depth and holistic. They mentioned that Ayurvedic consultations facilitated trust and confidence in the treatment process. Participants revealed that while Western Medical consultations for NCDs are more focused on quick, transactional encounters, Ayurveda offers a more comprehensive approach, prioritising the patient–practitioner relationship. Participants also revealed that Ayurvedic practitioners spent time discussing all health‐related issues during consultations. They described being heard and appreciated as Ayurvedic practitioners related to their individual needs on a personal level. This gave them a sense of partnership. Participants revealed that with Ayurveda, they not only received expert guidance but they also felt in control of their health journey and treatment experience.

‘Western medical practitioners don't have a lot of time, and a lot of attention to give, so it felt quite impersonal at times … with Ayurveda, I felt that the practitioners were more invested in me recovering from the symptoms, and they were able to give more time and attention’.

P1

##### 3.2.2.2 Integration of Western Diagnostics

Participants mentioned that Ayurvedic practitioners employed traditional Western physical examinations while managing their NCD condition. They revealed that Ayurvedic practitioners also incorporated modern diagnostic tools, like blood tests, alongside traditional assessments during diagnosis and clinical monitoring of NCDs. This combination appeared to increase trust in treatment efficacy.

‘My Ayurvedic practitioner would check my blood test to determine my thyroid levels, and it appeared to be balanced for a good while’.

P2

For many participants, the combination of Ayurveda's holistic lens with the diagnostic precision of Western medicine provided reassurance about both the credibility and the efficacy of their treatment. It also enabled them to benefit from both systems.

##### 3.2.2.3 Treatment Practices and Preferences

Participants used a range of Ayurvedic treatments—dietary modifications, lifestyle changes, herbal medications, and detoxifying and purifying therapies such as Panchakarma. They reported using a diverse range of Ayurvedic preparations for treating NCDs, consisting of commercially available Ayurvedic formulas and traditional concoctions. The preference for individual forms, such as tablets, powders and decoctions, depended on taste, ease of use and effectiveness. Some participants liked ready‐made medicines because they were easier to take, but others preferred conventional or raw herbs, feeling that these were more effective. Several participants reported adapting their routines to fit their daily schedules, using quicker options during the week, and engaging in more time‐consuming preparations when they had more flexibility. These choices reflected a balance between lifestyle practicality and perceived therapeutic benefit.

‘Over the years, I have tried various Ayurvedic treatments to manage NCDs. I have used ready‐made formulations available in tablet form, as well as powders that I had to prepare myself. For example, I have boiled powders to create a decoction or made them into a paste. Obviously, pills are just really easy, so I opt for the pill version’.

P9

##### 3.2.2.4 Perceived Health Improvements

Participants also described a wide range of perceived health improvements associated with their engagement with Ayurvedic care. The more general outcomes included improvements in the physical functioning of various organs of the body. Another shared finding was the belief that Ayurveda addresses the root cause of NCD rather than offering short‐term symptom suppression. In some cases, this translated into fewer flare‐ups of recurring conditions, improved daily mobility or more stable metabolic health. Several participants described Ayurvedic care as instrumental in preventing the need for invasive procedures or long‐term pharmaceutical use.

‘I take Ayurvedic supplements to help with osteoarthritis in my hips, and knees… I'm doing well and putting off joint replacements as long as possible’.

P11

Several participants described substantial improvements in internal health markers for various NCD conditions and bodily functions after sustained engagement with Ayurvedic care. These included reports on hormonal balance, metabolic regulation and resolution of long‐standing health challenges that had previously limited quality of life. Participants commonly attributed these changes to the combined effect of personalised herbal therapies, dietary modifications and lifestyle practices prescribed by Ayurvedic practitioners.

‘After following the plan for 6 months, my triglycerides went from 17 to 2 mmol/L’.

P2

#### 3.2.3 Challenges and Concerns in Accessing and Sustaining Ayurvedic Care

While Ayurveda was generally viewed positively, participants also identified several challenges related to safety, adherence, access and cost.

##### 3.2.3.1 Safety Concerns and Product Quality

Most participants had positive experiences with Ayurveda and Ayurvedic medicines for treating NCDs. However, some expressed concern about the safety and quality of some herbal products, particularly those obtained directly from India. Participants reported hearing or reading stories in the media about publicised information about heavy metal contamination in some Ayurvedic medicines used to treat NCDs. Some participants recalled that their perceptions of risk had been shaped by news articles or investigative reports highlighting instances of lead, mercury or arsenic in Ayurvedic medicines. Others considered first hand accounts from close friends or relatives who had used Ayurveda to manage NCDs, leading them to be more selective about where and how they obtained Ayurvedic medicines. Rather than purchasing from unknown or unregulated sources, participants emphasised the importance of consulting qualified Ayurvedic practitioners who could recommend reliable, quality‐assured products.

‘Generally, I find the treatments for NCDs to be good, but a word of caution, the herbs are very strong, and very important that the practitioner is well qualified. Another type of herb that has been somewhat controversial is mineral herbs because improper treatment could be dangerous’.

P4

Despite these concerns, trust in the practitioner's expertise and transparency in sourcing helped many participants feel reassured. They expressed greater confidence when Ayurvedic medicines used for NCDs were sourced through UK‐based practitioners or pharmacies that adhered to stricter safety standards.

‘I used to go and collect it at the centre in North London. However, during the COVID time, and so on, we started getting things posted, so we stayed with that. For now, I trust only in what I get posted to me; I request it on the phone, or by text, and it's sent off to me’.

P2

Overall, although safety concerns did not deter most participants from using Ayurvedic care, they influenced how participants approached product use, often reinforcing the importance of expert guidance and regulated sourcing.

##### 3.2.3.2 Adherence to Ayurvedic Medicines

Participants experienced different levels of challenges in adhering to Ayurvedic treatment for NCDs. Most participants were open to receiving Ayurvedic care. However, it was quite a challenge in practice to maintain such rigid regimes, especially in combination with ongoing Western medical treatments targeted at NCDs. Participants reported that the use of Ayurvedic medicines in tablet form was comfortable to adopt in their daily lives. However, they found compliance with the lifestyle and dietary precepts of Ayurveda even more challenging. Participants often struggled with the time, commitment and consistency required, especially when balancing work and family responsibilities. Many participants also described the emotional and social cost of adhering to Ayurvedic regimens for NCDs; there were times when dietary restrictions or treatment routines conflicted with social events, such as dining out or attending social gatherings.

‘I was told to follow a very strict routine for a certain number of months, and that was what I was told would bring me back to balance when managing my NCD condition. I did find it difficult to stick to the recommended path, and tend not to always follow exactly what they said, especially during social gatherings. I think it was the right advice, but for whatever reason, I didn't always manage to follow through very well on it’.

P1

Nevertheless, a significant number of participants managed to follow the Ayurvedic concepts for managing NCDs quite strictly, whereas the remainder opted for a flexible approach. Some participants mentioned that they adapted recommendations to better fit into their daily lives or that they tried to regularly check in with the practitioners to stay motivated and take responsibility.

##### 3.2.3.3 Difficulty in Sourcing Ayurvedic Medicines for NCD Management

Access to Ayurvedic medicines for the long‐term management of NCDs was a major concern for many participants. Whereas conventional Western medicines could often be purchased at a local pharmacy or prescribed by general practitioners, Ayurvedic medicines usually had to be obtained via more informal sources. This may be through personal preparation by an Ayurvedic practitioner, importing the medicine from abroad, or ordering online products. Purchasing Ayurvedic medicines was associated with issues such as availability, cost and trustworthiness. Participants managing NCDs reported that maintaining a consistent supply of specific Ayurvedic formulations was essential to their ongoing Ayurvedic care. They mentioned that shipping delays and local unavailability of Ayurvedic medicines meant that individuals who relied on specific herbs or preparations to manage NCDs had to source them online.

‘If the Ayurvedic pharmacy didn't have stock, I'd try to find it online, but it wasn't easy.’

P7

Some participants revealed that the difficulty in sourcing Ayurvedic medicines often posed a barrier to accessing them. They mentioned that this disruption, in some cases, led to periods of treatment interruption, non‐adherence or symptom relapse. A few participants who had been on Ayurvedic treatment for many years emphasised how they had to navigate these challenges over the years to maintain stable treatment plans. They mentioned the importance of treatment adherence, particularly when managing some NCD conditions where continuity is essential. These challenges highlight the need for more accessible and reliable channels to obtain Ayurvedic treatments in the UK context.

##### 3.2.3.4 Financial Challenges in Accessing Ayurvedic Care for NCDs

The cost associated with Ayurvedic care was emphasised as a barrier for most participants, especially those who used it as a long‐term approach to managing their NCDs. Participants revealed that, compared with standard Western treatments, Ayurvedic consultations and drugs are not covered by the NHS. Therefore, participants had to bear the sole cost for sustained use, posing a considerable financial commitment, particularly for those with low incomes. Some participants mentioned that although they could afford the initial consultation and short‐term herbal medicine prescriptions, the overall costs of maintaining a full Ayurvedic programme, especially when it involved regular follow‐up appointments, lifestyle counselling, specialist detox therapies (like Panchakarma) and imported herbal products, proved to be a significant financial burden.

‘A couple of years ago I started taking Ayurveda, but it is so expensive I just had to stop it because I couldn't afford it. I stopped it and then went back to the conventional medicine and that made my disease worse. It should be on the NHS; I think that people should be given the option to choose Ayurvedic medicine rather than take on conventional medicine and get nowhere’.

P18

For participants who tried to combine Ayurvedic and conventional medical treatments, this was particularly challenging, as the Ayurvedic approach was often used inconsistently or discontinued early. For those whose conditions required regular intervention, a lack of financial support meant difficult choices: whether to reduce reliance on Ayurvedic components of care, look for cheaper and possibly less effective alternatives, or focus instead on Western medical treatments for which they had greater access in public health facilities. Notwithstanding these limitations, many participants favoured introducing Ayurveda into the NHS, or some kind of public subsidy system, considering this an important movement towards greater diversity and access to more holistic, prevention‐based care. Some participants compared it to other complementary therapies, such as acupuncture, which are gradually being more broadly adopted by the NHS.

‘I think it's a good thing if Ayurveda can be included in the NHS. I read a little about acupuncture, and other things that are happening inside the NHS, and obviously all the complementary therapies like CBT…. I think it's great’.

P9

## 4 Discussion

This study provided insights into the experiences and perspectives of people with NCDs regarding the use of Ayurveda for managing their conditions. One of the most significant findings was the highly personal and culturally rooted motivations for seeking Ayurvedic treatment. Several findings from this study aligned with existing literature on Ayurveda use among individuals with NCDs in Sweden and Germany [25, 26]. The results were also consistent with a qualitative systematic review on the use of Ayurveda for NCD management in OECD countries [27]. Personal motivations for using Ayurveda included family tradition, cultural congruence and personal values that were compatible with Ayurveda's holistic principles [10, 27]. These results supported anthropological discussions of ‘health cultures’, in which personal belief systems and socio‐cultural contexts played a significant role in health‐seeking behaviour [28, 29, 30]. For most participants, Ayurveda was not simply viewed as an alternative to Western medicine but as a more natural and philosophically congruent system of healing‐supporting existing literature that suggests CAM users often seek care aligned with their worldview [10, 31].

These motivations reflected broader processes of health‐related identity construction [10, 28, 29, 30, 31]. This study contributed to current understandings by showing how individuals in the United Kingdom navigated NCD management through a pluralistic health landscape [32, 33]. Positioning these findings in the context of medical sociology, anthropology and global health contexts allowed a more nuanced understanding of the intersection of structural dynamics, cultural identity and individual autonomy in the treatment of NCDs [33, 34, 35, 36, 37, 38]. These dynamics were particularly evident among participants of South Asian heritage, whose use of Ayurveda was shaped not only by personal health beliefs but also by deep‐rooted ties to diasporic identity and shared cultural heritage [39]. For these individuals, engagement with Ayurveda extended beyond symptom management to include acts of cultural continuity and identity formation.

These findings indicated transnational health‐seeking, in which Ayurveda served as a modality of healing as well as a tool for maintaining diasporic connections and navigating global health terrains [33, 35, 36, 40]. These insights suggest the necessity to reconceptualise health‐seeking behaviour, not merely as a response to disease, but as a dynamic practice shaped by personal values and cultural worldviews.

In our study, very few participants made the decision to use Ayurveda due to a single factor. Rather, these decisions were shaped by a constellation of factors—personal, cultural and epistemic. As in previous studies, dissatisfaction with the Western medical approach to NCD management was a major reason for seeking care, although not the only one [27]. Participants actively sought care that was more philosophically congruent and culturally relevant. Ayurveda, with its emphasis on balance, interconnectedness and prevention, offers a healing language that resonates with their lived realities [33, 34, 37].

Participants also reported resonating with Ayurveda's philosophy of self‐repair. Such narratives align with previous literature suggesting that health‐seeking behaviours are embedded in systems of meaning and that individuals seek care not merely to relieve symptoms but to restore coherence to disrupted lives caused by various NCDs [33, 34, 35, 36, 37, 38]. This emphasis on personal responsibility and empowerment resonates with an understanding of health citizenship, which promotes the idea that individuals are to become agents with regard to their own health [33, 35, 36, 40]. Nonetheless, this empowerment was not without its challenges. The requirement to maintain complicated regimens for managing NCDs placed a substantial demand on participants, particularly those with limited systemic support.

Despite their commitment to Ayurvedic principles and practices, the participants faced significant structural and financial barriers to accessing Ayurvedic care in the United Kingdom. The most common barriers were cost, local availability and lack of NHS integration. Several participants expressed frustration at having to pay out of pocket, often at significant personal expense, to access what they considered a fundamental part of their well‐being [9]. These findings addressed the larger problem of inequity in health as it relates to access to CAM [8, 9, 35]. While medical pluralism may exist in theory, in practice it often reproduces social exclusions, privileging those with the economic and cultural capital to navigate alternative systems [32, 35]. These limitations were indicative of larger political and economic factors which influence the use of alternative therapies in the United Kingdom [32, 33, 35, 36, 41, 42, 43]. A more critical perspective is that the marginalisation of CAM in Western public health mirrors more fundamental ideological commitments to Western medical hegemony and the commercialisation of health governance [32, 33, 37].

Although participants did not directly raise concerns about product safety, it is a significant issue relating to the practice of Ayurveda in the United Kingdom. Many participants presumed that Ayurvedic products purchased from reputable practitioners or established retailers were safe. However, reports from previous studies have shown that some imported Ayurvedic medicines may include impurities such as heavy metals [44, 45]. In the absence of an official UK regulation on the manufacture, import and sale of Ayurvedic products, ensuring safety remains difficult. Therefore, future research and policy development should consider the possible health risks of these products to support patients in making safe, informed choices [44, 45].

Participants expressed a strong desire for the integration of Ayurveda into the NHS. However, these views reflect personal aspirations rather than established evidence on safety, clinical effectiveness or cost‐effectiveness. As this study focused on participants' experiences and perspectives, we did not evaluate the clinical or policy feasibility of integration. We acknowledge that robust evidence on the safety and effectiveness of Ayurvedic treatments in UK settings is limited. Further research is needed to assess the practical implications of integrating Ayurveda into mainstream healthcare before making any policy or service‐level decisions.

## 5 Strengths and Limitations

This is the first qualitative study to explore the use of Ayurveda by people with NCDs in the United Kingdom. This study made a theoretical contribution to the anthropological and sociological debates on medical pluralism, care ethics and the role of traditional medical systems, such as Ayurveda, in modern healthcare contexts [32, 33, 35, 36, 38]. It provides empirical evidence in support of the view that the use of CAM medicines like Ayurveda in NCDs management is embedded in cultural narratives, in personal ideas of what healing means [33, 34]. It also demonstrates how Ayurveda's position at the intersection of care and health governance raises important questions about legitimacy, access and authority within pluralistic health landscapes [37]. Despite the study being conducted in the United Kingdom, the findings may hold relevance for other multicultural health environments.

The study was conducted using semi‐structured interviews, with themes derived inductively from participants' narratives [19]. The study, while influenced by medical sociology [10] and anthropology, remained firmly based on the evidence [19, 33, 34, 38]. A limitation is that interviews were conducted online instead of in person, potentially restricting the monitoring of nonverbal signs. Nonetheless, online interviews can still produce substantial data and offer more flexibility [46].

One notable limitation relates to the recruitment strategy. Most participants were highly educated British Asian women, which limits the transferability of our findings. However, to our knowledge, this is also one of the first qualitative studies to explore the use of Ayurveda within the UK context, and little is known about the makeup of the population currently using this medicine. Ayurveda has its origins in South Asia, and social and cultural familiarity may partly explain why British Asian groups are more likely to seek out such care. At the same time, because Ayurveda is not available free of charge in the United Kingdom, its uptake may also be skewed towards individuals from higher‐income or more advantaged socio‐economic backgrounds. Although our findings provide insight into this user group, our results may not necessarily apply to the wider UK population or other ethnic groups [47, 48]. This highlights the contribution of the present study in filling a gap in available literature, and the need for future research, including participants from other subgroups [49, 50, 51].

This study reflected the participants' views; the authors have also conducted a separate study exploring the perspectives of Ayurvedic practitioners and have prepared a manuscript based on those findings, which has been submitted for publication elsewhere. Subsequent research involving policymakers and Western medical professionals could provide a more comprehensive understanding of Ayurveda's role in UK healthcare.

## 6 Conclusion

People living with NCDs described Ayurveda as a more natural and philosophically congruent healing system, reflecting their cultural and personal perspectives. Despite structural and financial challenges, they considered it a relevant option for managing their conditions. These findings suggest that Ayurveda continues to hold significance as a complementary approach to NCD management in the United Kingdom.

## Author Contributions

**Patricia Egwumba:** conceptualisation, investigation, formal analysis, methodology, validation, writing – original draft, writing – review and editing. **Kaushik Chattopadhyay:** conceptualisation, supervision, methodology, validation, writing – review and editing. **Laura Nellums:** supervision. **Manpreet Bains:** supervision, formal analysis, methodology, validation, writing – review and editing.

## Disclosure

The funding source was not involved in study design, data collection, analysis or interpretation, or in the writing of this manuscript.

## Ethics Statement

This study was performed in line with the principles of the Declaration of Helsinki. Approval was granted by the University of Nottingham's Faculty of Medicine and Health Sciences Research Ethics Committee (FMHS 298‐0523).

## Consent

Informed consent was obtained from all individual participants included in the study.

## Conflicts of Interest

The authors declare no conflicts of interest.

## Data Availability

All data supporting the findings of this study are available within the paper.
